# From theory to practice: New innovations and their application in conservation biology

**DOI:** 10.1002/aps3.11599

**Published:** 2024-05-31

**Authors:** Christopher P. Krieg, Carrie M. Tribble, Randall Long

**Affiliations:** ^1^ Department of Botany University of Wisconsin Madison Wisconsin USA; ^2^ School of Life Sciences University of Hawai'i at Mānoa Mānoa Hawai'i USA; ^3^ Department of Biology Lewis & Clark College Portland Oregon USA

Evolution has generated an extraordinary diversity of life on Earth that drives the function of natural ecosystems (Xu et al., 2020), human cultures (Clark et al., 2014), and economies (Hanley and Perrings, 2019; Paul et al., 2020). Plants are the most dominant life form on Earth (Bar‐On et al., 2018) and the decline of plant diversity has caused drastic shifts in natural ecosystems (Pugnaire et al., 2019), resulting in a loss of hundreds of billions of dollars (USD) per year from the global economy (Austin et al., 2020; Diagne et al., 2021). Plant species face unprecedented challenges to their survival, growth, and reproduction due to numerous threats, including anthropogenic factors such as land‐use change, habitat destruction, climate change, and illegal poaching (IUCN, 2023). The most urgent threats vary by region and species; thus, addressing individual threats to species survival worldwide will require broad knowledge of plant organismal function, ecology, and evolution and the creation of innovative, targeted tools and applications. This special issue features new techniques and approaches across multiple disciplines (from molecules to ecosystems) and scales of inquiry (from individual plants to global perspectives), with a central focus on the development and dissemination of new methods and perspectives in conservation biology.

Conservation biologists and practitioners around the globe are conducting research and enacting policies to conserve and preserve plant diversity at the local and regional scales. One primary obstacle to the conservation of plant diversity at larger scales has been the lack of tools that directly aid the coordination of research efforts and knowledge from around the world. Linsky et al. (2024) present a new collaborative framework called the Global Conservation Consortia (GCC). Under this framework, researchers and practitioners are united by a shared focus on a specific ecological or taxonomic group (e.g., trees, cycads, magnolias, oaks) to develop comprehensive conservation action plans that scaffold efforts across local, regional, and global scales. While gathering and collating data from around the globe has historically been a challenge, a new tool hosted by the Botanic Gardens Conservation International (BGCI), reported here by Quintana et al. (2024), aims to close knowledge gaps on the conservation status of threatened tree species across regions, increase collaboration, and provide information to decision‐makers. The Conservation Action Tracker, part of the GlobalTree Portal, gathers information about the current status of threatened species, action/recovery plans, ex situ collections, species protections, and policy and outreach programs. This online database is freely available so that it can be used to guide conservation efforts and monitor their success. It allows for comparisons between different species and regions, and importantly, highlights the areas where conservation efforts are deficient. Large‐scale databases and collaborative networks like GCC and BGCI are key developments that can improve the effectiveness of species knowledge dissemination among conservation researchers and practitioners.

A main goal of conservation networks like the GCC and BGCI is to create and expand metacollections of living plants that are of high conservation value. The preservation of wild diversity in ex situ (meta)collections is one of the most effective tools in conservation biology. Thus, understanding the extent to which current collection practices capture the biological diversity of wild populations is critical to their effectiveness, and strategies to improve the diversity represented in ex situ collections are of extreme interest to conservation biologists and practitioners alike. Linan et al. (2024) describe an effective approach to assess the genetic diversity of ex situ and in situ individuals of the tropical tree *Karomia gigas*, of which fewer than 100 individuals remain on Earth. Using a genetic approach, the authors were able to determine the relative proportion of individuals that share kinship and thus better inform breeding and outcrossing approaches to preserve the genetic diversity of ex situ collections. Similarly, Rosenberger and Hoban (2024) used computer simulations to understand better the contribution of propagule dispersal and population structure to the distribution of genetic diversity among maternal individuals. These empirical and theoretical data demonstrate the importance of understanding the diversity of ex situ populations relative to in situ populations to improve the effectiveness of ex situ (meta)collections for preserving extant plant species.

One of the most effective practices for reversing diversity loss is species and/or habitat restoration, which is often performed with source material from ex situ collections. However, many restoration efforts are unsuccessful, and improving the success of restoration efforts could transform conservation practices broadly. Prakash et al. (2024) demonstrate the importance of considering source population genetic diversity when maximizing outplanting diversity and evolvability in *Picea rubens*, a declining forest tree in eastern North America. By integrating genomic data, field experiments, and boots‐on‐the‐ground conservation, the authors demonstrate a positive association between combined source populations of outplanted individuals, early‐life fitness, and evolvability. Despite advances made in conservation research, there is a current gap in sharing the outcomes of restoration efforts of plants due to a disconnect between conservation research and conservation practice. Bellis et al. (2024) introduce a database of reintroductions within the United States, called the Center for Plant Conservation Reintroduction Database (CPCRD), to enable the sharing of restoration efforts and their progress and outcomes. The CPCRD collaboration offers great promise to facilitate better management decisions by restoration practitioners, as well as encourage scientific engagement by providing a long‐term database.

A primary limitation for restoration practitioners working in seed‐plant systems is collecting and identifying seeds of both rare and common species. Currently available methods are expensive and time‐consuming, using either expensive machines that can separate size‐ or shape‐specific seeds one at a time, or the time of highly skilled individuals. Multi‐year projects also often introduce user error if the same individuals are not responsible for sorting each season. Reek et al. (2024) tested a novel way to sort conifer seeds using image recognition and machine learning. After training their model on a set of conifer seeds, they validated it against manual identification and found it to be similar in accuracy. This new method of seed counting was more efficient and, if it can be applied to other species, will provide time and cost savings in conservation efforts to monitor the seed availability of various species.

Predicting extinction risk and possible future changes to various aspects of species' biology (e.g., distribution, phenology, reproductive success, physiological performance, gene expression) before they require intensive restoration efforts has become a cornerstone of conservation biology research. For example, the Evolutionarily Distinct and Globally Endangered (EDGE) and EDGE2 metrics are two recent approaches that integrate extinction risk and the evolutionary distinctiveness of geographic areas to identify conservation priorities. Pizzardo et al. (2024) compared the performance of these metrics with more traditional metrics (e.g., phylogenetic diversity) to determine conservation prioritization using a tropical legume clade (*Chamaecrista* ser. *Coriaceae)* found in the Brazilian campo rupestre ecosystem. While EDGE and EDGE2 both incorporate extinction risk, Pizzardo et al. draw attention to their requirement for complete phylogenetic data, which may be unrealistic for data‐deficient regions or taxonomic groups.

In contrast to broad‐scale approaches that rely on phylogenetic diversity to identify conservation priorities, species distribution models (SDMs) are commonly used to predict potential changes in species ecology and distribution into the future. Evolving the use of these models by integrating different types of geographic, biological, and historical data is a core aim in conservation biology to improve the predictability of species outcomes in the future. Hansen et al. (2024) tested the performance of correlative SDMs for the invasive aquatic plant European frog‐bit using data at different geographic scales and with and without including field observations. The authors found that interpretations of model outputs can be very different based on the data types included, including which factors are driving species distributions. These comparisons show the direct implications of data integration on the perception of species biology and thus inform or misinform aspects of species biology that are of conservation concern. Similarly, Edwards‐Calma et al. (2014) integrated historical land‐use data with a recently developed statistical model for estimating a hypothetical fundamental niche for naturalized and native species of ferns. The authors make robust comparisons of species niche occupancy in light of historical land‐use changes that reveal the portions of native fern niches that are occupied by naturalized species and discuss the implications for biotic interactions to limit species distributions. These studies demonstrate the power of integrating different data types to improve the conservation value of SDMs and to predict changes in aspects of species biology across space and time.

The new methods and approaches presented in this special issue speak to the diversity of approaches that botanists are employing for their work to preserve threatened species and ecosystems in the face of a biodiversity crisis worldwide. Many of these papers were originally presented at the symposium “From Theory to Practice: New Innovations and Their Application in Conservation Biology,” held at the Botany 2023 meeting; the symposium had two goals: (1) to highlight new technologies and novel uses of data sets to document and promote the conservation of plant species, and (2) the celebration of the 10th anniversary of *Applications in Plant Sciences* as a publication of the Botanical Society of America. This special issue—which grew out of the 2023 symposium—highlights some of those advances, among others, to aid in regional and worldwide conservation efforts, including more collaborative data repositories, genetic techniques, machine learning, and refined models. As conservation researchers and practitioners continue to innovate and develop new tools to prevent the further loss of biodiversity, it will be important to continue to share and promote these applications to the wider community. We hope that this special issue will be a useful resource for the community as it continues to innovate and solve problems while facing the challenges ahead.

## AUTHOR CONTRIBUTIONS

C.P.K. and R.L. initiated this special issue, and C.M.T. contributed to its development. All authors contributed to editorial duties for the manuscripts included in this special issue. All authors contributed text for the manuscript, and C.P.K. combined those contributions and led the writing and editing. All authors approved the final version of the manuscript.
